# Chronotype: A Tool to Screen Eating Habits in Polycystic Ovary Syndrome?

**DOI:** 10.3390/nu14050955

**Published:** 2022-02-23

**Authors:** Luigi Barrea, Ludovica Verde, Claudia Vetrani, Silvia Savastano, Annamaria Colao, Giovanna Muscogiuri

**Affiliations:** 1 Dipartimento di Scienze Umanistiche, Università Telematica Pegaso, Via Porzio, Centro Direzionale, Isola F2, 80143 Napoli, Italy; luigi.barrea@unina.it; 2 Centro Italiano per la Cura e il Benessere del Paziente con Obesità (C.I.B.O), Endocrinology Unit, Dipartimento di Medicina Clinica e Chirurgia, Federico II University, Sergio Pansini, 5, 80131 Naples, Italy; ludoverde96@gmail.com (L.V.); sisavast@unina.it (S.S.); acolao@unina.it (A.C.); 3 Endocrinology Unit, Dipartimento di Medicina Clinica e Chirurgia, Federico II University, Sergio Pansini, 5, 80131 Naples, Italy; c.vetrani@libero.it; 4 Cattedra Unesco “Educazione alla Salute e allo Sviluppo Sostenibile”, Federico II University, 80131 Naples, Italy

**Keywords:** PCOS, chronotype, Mediterranean diet, diet, nutrition, obesity, lifestyle

## Abstract

Polycystic ovary syndrome (PCOS) is the most common endocrine disorders in women of reproductive age, whose lifestyle approach is an essential part of the treatment. Recently, chronotype, i.e., a trait that determines individual’s circadian preference in behavioral and biological rhythms, has been reported to play a role in determining nutrition preferences and the risk of developing chronic diseases. Thus, the aim of this study was to investigate if chronotype categories (morning, evening, and neither) could be used as tool to screen eating habits in women with PCOS. In this observational cross-sectional study, we assessed anthropometric measurements, lifestyle habits, chronotype categories, adherence to the Mediterranean Diet, dietary pattern, and metabolic parameters in 112 women with PCOS. Chronotype was classified as morning in 27.7%, evening in 42.9%, and neither in 29.5% of subjects. Women with PCOS with evening chronotype showed significantly higher percentages of grade I (*p* = 0.003) and grade II obesity (*p* = 0.001), did less regular exercise (*p* < 0.001), and most of them were smokers (*p* < 0.001) compared to those with neither and morning chronotypes. Women with PCOS with evening chronotype were significantly more insulin resistant (Homeostatic Model Assessment of Insulin Resistance (HoMA-IR) cut off > 2.5) than other two chronotypes (*p* < 0.001). Women with PCOS with evening chronotype had the lowest PREvención con DIetaMEDiterránea (PREDIMED) score, consumed more calories (*p* < 0.001), total (*p* < 0.001) and simple carbohydrates (*p* < 0.001), total fat (*p* < 0.001) and saturated fatty acids (*p* < 0.001), polyunsaturated fatty acids (*p* < 0.001) and n-6 polyunsaturated fatty acids (*p* < 0.001), and less fiber (*p* < 0.001) than women with PCOS with other chronotypes. In addition, women with PCOS with evening chronotype consumed less extra virgin olive oil (*p* = 0.001), legumes (*p* = 0.038), fish/seafood (*p* < 0.001), and tree nuts (*p* = 0.041) than women with PCOS of the other two chronotype categories and less red wine (*p* < 0.001) and more red/processed meat (*p* < 0.001) than women with PCOS with morning chronotype. In conclusion, in women with PCOS, evening chronotype has been associated with a most severe insulin resistance and unhealthiest eating habits. Thus, chronotype assessment could be an effective tool to screen the eating habits, and more generally the lifestyle, of women with PCOS.

## 1. Introduction

Polycystic ovary syndrome (PCOS) is one of the most common endocrine disorders in women of reproductive age reaching a prevalence rate of 5–10% [1]. Distinctive features include chronic anovulation, biochemical and/or clinical hyperandrogenism and polycystic ovarian morphology [2]. Diagnosis of this syndrome is based on the Rotterdam criteria (2003), when 2 out of 3 criteria are satisfied, while other etiologies are excluded [2]. Hyperandrogenism is one of the main features of PCOS and evidence supports that it is one of the most used markers to assess severity of PCOS [3]. In addition, obesity, low-grade chronic inflammatory state and insulin-resistance (IR) often coexist in women with PCOS [4]. Furthermore, we also previously showed that women with PCOS had different body composition compared to women without PCOS, with the lowest values of fat free mass and phase angle (PhA), a marker of the chronic inflammatory state [5]. Beyond genetic predisposition, several pathogenetic factors could be involved in the pathogenesis of PCOS and PCOS-related complications [6,7]. In the management of women with PCOS, the crucial role of lifestyle and in particular of nutrition is well known, and it is currently at the forefront of treatment of this condition [8,9]. However, a tailored nutritional approach is based on an accurate assessment of eating habits at the baseline. The food diary is a useful tool, but it is time-consuming, and it is filled by the patients that may not objectively report eating habits. Thus, in order to drive the nutritional approach, there is the need to find an easy and valid tool to screen eating habits of PCOS in the nutrition outpatient clinic. Chronotype, i.e., the attitude of a subject determining individual circadian preference in behavioral and biological rhythms related to the external light–dark cycle, has been reported to play a role in determining nutrition preferences and the risk of developing chronic diseases [8,9,10,11]. There are three categories of chronotype: morning chronotype (defined “lark”) tends to wake up early and prefers activities earlier in the day, while the evening chronotype (defined “owl”) generally wakes up later and prefers to perform his/her main activities in the late afternoon or evening [10]. Neither chronotype is in an intermediate position between the morning and evening chronotypes [10]. Evening chronotype has been associated with a low adherence to the Mediterranean Diet (MD) [12], obesity [13], and IR [14], all of which are often present in women with PCOS [15].

Based on this background, this observational cross-sectional study aimed to investigate if chronotype categories (morning, evening, and neither) could be used as tool to screen eating habits in women with PCOS in order to drive nutritional approach.

## 2. Materials and Methods

### 2.1. Design and Setting

This cross-sectional, observational study was carried out in women with PCOS attending the Unit of Endocrinology, Department of Clinical Medicine and Surgery, University Federico II of Naples (Italy), from January 2015 to September 2021. The study was approved by the local ethics committee and carried out in accordance with the Declaration of Helsinki for human experimentation. The aim of the study was clearly explained to all study participants and written informed consent was obtained.

### 2.2. Population Study

After obtaining written informed consent, 300 women with PCOS (aged 18–39 years) were consecutively enrolled. To increase the homogeneity of the patient sample, we included only treatment-naïve women with PCOS. Patients with a diagnosis of PCOS according to the European Society for Human Reproduction and Embryology/American Society for Reproductive Medicine (ESHRE/ASRM) classification were eligible [2]. This includes the presence of two of the three features hyperandrogenism (either clinical (hirsutism by elevated Ferriman–Gallwey score) or biochemical (elevated testosterone or free androgen index), oligomenorrhea (interval between two menstrual periods more than 35 days), or amenorrhea (no vaginal bleeding for at least six months) and the presence of polycystic ovaries on ultrasound (≥12 follicles 2–9 mm in diameter or an ovarian volume > of 10 mL in at least one ovary) [2]. The inclusion criteria were: premenopausal women, aged 18–39 years and Body Mass Index (BMI) ranged from 21.0 to 39.9 kg/m^2^.

Criteria for exclusion from the study were: age <18 and ≥40 years; menopause (defined as amenorrhea for ≥3 years or amenorrhea for ≥1 but <3 years and plasma follicle-stimulating hormone concentrations elevated into the postmenopausal range); pregnancy or lactation in the last 6 months (15 women); not ovary-related hyperandrogenism and/or biochemical hyperandrogenemia, oligomenorrhea due to secondary causes according to the Endocrine Society Clinical Practice Guidelines and previous publications, including endocrine disorders (congenital adrenal hyperplasia, androgen-secreting tumors, Cushing’s syndrome, hyperprolactinemia, thyroid dysfunction, and adrenal disorders) (3 women) [16]; metabolic disorders such as type 2 diabetes (12 women), hypertension (32 women); diagnosed anemia (23 women), or other metabolic disorders requiring special diets (3 women); preexisting systemic or psychiatric diseases (8 women); use of medications that affect carbohydrate or lipid metabolism (oral contraceptives, metformin, antiepileptic drugs, antipsychotics, statins, and fish oil) (24 women); special diet plans or hypocaloric diet in the past 3 months, including vegan or vegetarian diet (41 women); supplementation with antioxidants, vitamins, or minerals (19 women); occasional or current use of medications that may affect fluid balance, including nonsteroidal anti-inflammatory drugs, diuretics, laxatives, corticosteroids, and/or antiandrogenic corticosteroids (8 women).

**Figure 1:**
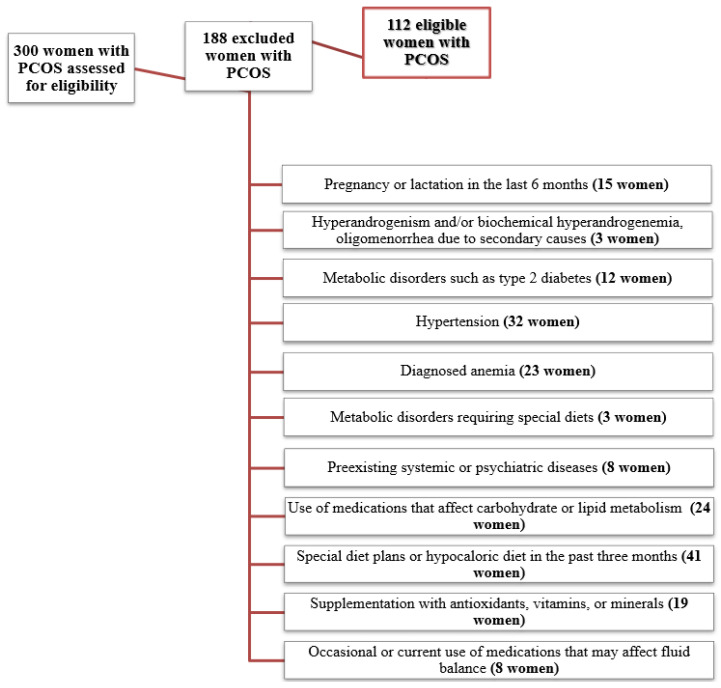
Flow chart of the studied women with PCOS.

Therefore, a total of 112 women with PCOS remained for analysis. The flow diagram of the studied women is shown in Figure 1.

### 2.3. Sample Size Justification and Statistical Power

The calculation of the sample size was performed a priori as, to our knowledge, there are no similar clinical studies in the literature. The calculation was carried out considering an effect size of 0.8 with a type I error of 0.05 and a power of 90% using G Power Software. The number of subjects to be enrolled was found to be 34 per group. As our sample not only reached the necessary number of subjects, but also strengthened the power of results the statistical analysis, we decided to include all of them.

### 2.4. Lifestyle Habits, Nutritional Assessment, and Anthropometrical Assessment

We defined individuals who smoked at least one cigarette per day as current smokers, individuals who had quit smoking at least one year before the survey as former smokers, and the remaining participants as non-smokers. Participants who exercised at least 30 min per day (YES/NO) were defined as physically active, as we have reported in detail in previous studies [16,17,18]. The adherence to the MD was evaluated using the 14-items questionnaire for the assessment of PREvención con DIetaMEDiterránea (PREDIMED) [19], as previously reported [20].

From seven-day food records, we obtained the dietary histories of participants by a face-to-face interview with a nutritionist, as previous reported [5]. The total energy intake and the quantities of macronutrients has been calculated based on seven-day food records. Anthropometric measurements and biochemical and hormonal parameters were taken between 8 a.m. and 12 noon. All subjects were measured after an overnight fast. Anthropometric measurements were performed by the same operator (a nutritionist with experience in nutrition and body composition assessment) according to the International Society for the Advancement of Kinanthropometry (ISAK 2006). All anthropometric measurements were performed with subjects wearing only light clothing and no shoes, as previously reported [18,21,22]. Height was assessed to the nearest 0.5 cm using a wall stadiometer (Seca 711; Seca, Hamburg, Germany). Body weight was measured to the nearest 0.1 kg using a calibrated beam scale (Seca 711; Seca, Hamburg, Germany). In accordance with the World Health Organisation, BMI was classified as follows: normal weight: 18.5–24.9 kg/m^2^, overweight, 25.0–29.9 kg/m^2^; grade I obesity, 30.0–34.9 kg/m^2^; grade II obesity, 35.0–39.9 kg/m^2^ [23]. Waist circumference (WC) was measured to the nearest 0.1 cm with a non-extendable tape measure at the natural indentation or halfway between the lower edge of the rib cage and the iliac crest if no natural indentation was visible, according to the National Center for Health Statistics [24].

### 2.5. Assay Methods

Collection of samples was done between 8 and 10 am following an overnight fast of at least 8 h and stored at −80 °C until processing in the central biochemistry laboratory of our institute. Plasma glucose was analyzed using a Roche modular analytical system while fasting insulin levels were measured using a solid-phase chemiluminescent immunoassay. The Homeostasis Model Assessment of Insulin Resistance (HoMA-IR) was calculated according to Matthews et al. [25]. HoMA-IR value > 2.5 was used as cutoff value for the IR. Intra- and inter-assay values CV were <7% for all assays performed.

### 2.6. Chronotype Assessment

To assess the participants’ chronotype, we used the Morningness–Eveningness Questionnaire (MEQ) [10]. The MEQ consists of 19 multiple-choice questions regarding sleep habits and daily performance, such as when feeling most productive in physical or mental activities, when feeling most tired, and when feeling most energetic. The sum of the individual items produced a total score ranging from 16 to 86 and based on their score, individuals were classified as morning (59–86), neither (42–58), or evening (16–41) chronotypes [10].

### 2.7. Statistical Analysis

The data distribution was evaluated by Kolmogorov–Smirnov test and data not normally distributed were normalized by logarithm. The chi square (χ^2^) test was used to determine the significance of differences in frequency distribution of BMI categories, physical activity, smoking habit, and HoMA-IR. An ANOVA test followed by the Bonferroni post hoc test was used to evaluate the differences between three groups. The correlations between study variables were performed using Pearson *r* correlation coefficients. A partial correlation was performed to control for effects of confounding factors on chronotype. Proportional Odds Ratio (OR) models, 95% Interval Confidence (IC), and R^2^ were used to assess the associations among chronotype score with single items of PREDIMED questionnaire in women with PCOS. Data were collected and analyzed using the MedCalc^®^ package (Version 12.3.0 1993-2012-Mariakerke, Belgium).

## 3. Results

One hundred and twelve women with PCOS (aged 24.21 ± 5.47 years; BMI 30.95 ± 5.66 kg/m^2^) were enrolled. Specifically, 24 subjects (21.4%) of them were normal weight, 29 subjects (25.9%) were overweight, and 24 subjects (21.4%) and 35 subjects (31.3%) had grade I and II obesity, respectively. The chronotype categories were distributed among women with PCOS as follows: 31 women (27.7%) morning, 33 women (29.5%) neither, and 48 women (42.9%) evening chronotype. BMI categories, lifestyle habits, and HoMA-IR of women with PCOS, according to chronotype categories, are reported in Table 1. Women with PCOS with evening chronotype showed significantly higher percentages of grade I (*p* = 0.003) and grade II obesity (*p* = 0.001) and were less prone to follow healthy lifestyles compared to those with neither and morning chronotypes; indeed, they did less regular exercise (*p* < 0.001) and most of them were smokers (*p* < 0.001). Evening chronotype also was significantly associated with a higher prevalence of subjects with HoMA-IR cut off > 2.5 than neither and morning chronotypes (*p* < 0.001).

Differences in age, BMI, WC, HoMA-IR, and adherence to the MD were showed in Table 2. There were no differences in age among the chronotype categories (*p* = 0.763). Finally, women with PCOS with evening chronotype had the lowest PREDIMED score, i.e., they had the lowest adherence to the MD compared to the other chronotypes (*p* < 0.001).

**Figure 2:**
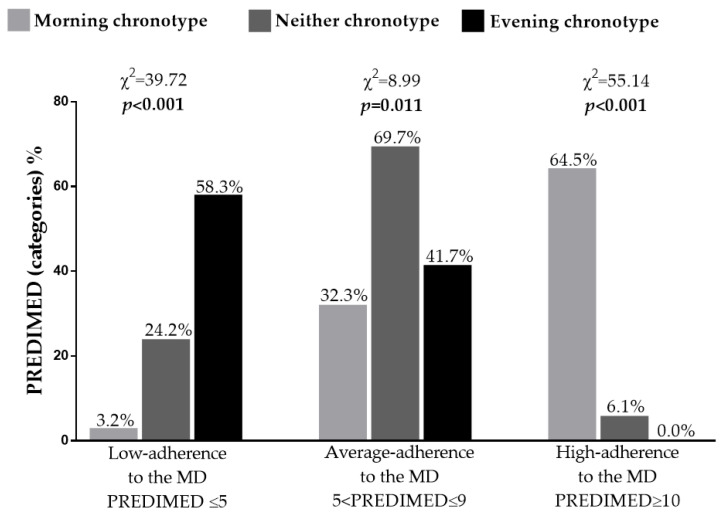
Adherence to the MD according to chronotype categories in women with PCOS. A *p* value in bold type denotes a significant difference (*p* < 0.05). MD, Mediterranean diet; PREDIMED, Prevención con Dieta Mediterránea.

Figure 2 reported adherence to the MD in women with PCOS grouped based on chronotype categories. Women with lower adherence to the MD presented the high percentage of evening chronotype (58.3%; *p* < 0.001). Of note, no women with PCOS with evening chronotype reported high adherence to the MD (0.0%; *p* < 0.001).

Table 3 reported differences in nutritional parameters of women with PCOS, according to chronotype categories. In detail, women with PCOS with evening chronotype consumed more total energy intake (*p* < 0.001), total and simple carbohydrates (*p* < 0.001), total fat, saturated fatty acids (SFA), polyunsaturated fatty acids (PUFA, and n-6 PUFA (*p* < 0.001 for all), and less fiber (*p* < 0.001) than women with PCOS with other chronotype categories.

Analyzing the response frequency of dietary components included in the PREDIMED questionnaire in detail, we found that, when compared to the other two chronotype categories, women with PCOS with evening chronotype consumed less extra virgin olive oil (EVOO) (*p* = 0.001), legumes (*p* = 0.038), fish/seafood (*p* < 0.001), and tree nuts (*p* = 0.041). In addition, women with PCOS with evening chronotype consumed less red wine (*p* < 0.001) and more red/processed meat (*p* < 0.001) than women with PCOS with morning chronotype (Table 4).

Correlation analyses were performed to investigate the association of chronotype scores with anthropometric measurements, metabolic, and nutritional parameters as reported in Table 5. Chronotype score was inversely associated with BMI (r = −0.775; *p* < 0.001), WC (r = −0.674; *p* < 0.001), fasting plasma glucose (r = −0.486; *p* < 0.001), insulin levels (r = −0.568; *p* < 0.001), HoMA-IR (r = −0.556; *p* < 0.001), and positively associated with PREDIMED score (r = 0.803; *p* < 0.001). In addition, the chronotype score was also inversely associated with total energy intake (r = −0.507; *p* < 0.001), total carbohydrate (r = −0.563; *p* < 0.001), simple carbohydrates (r = −0.609; *p* < 0.001), total fat (r = −0.598; *p* < 0.001), SFA (r = −0.711; *p* < 0.001), unsaturated fat (r = −0.324; *p* < 0.001), PUFA (r = −0.465; *p* < 0.001), and n-6 PUFA (r = −0.456; *p* < 0.001) and positively associated with fiber intake (r= 0.531; *p* < 0.001).

Table 6 showed the results of the bivariate proportional OR model performed to assess the association of chronotype score with food items of the PREDIMED questionnaire. EVOO, vegetable, fruit, fish, poultry, nuts, and wine consumption were positively associated with chronotype score, while the highest OR of soda drinks, red meats, butter, cream, margarine commercial sweets and confectionery seemed to have a negative effect on chronotype score.

## 4. Discussion

Women with PCOS and evening chronotype had higher rates of obesity and were less prone to follow healthy lifestyle. In fact, they exercised less regularly and smoked more than women with PCOS with neither and morning chronotypes.

In agreement with our results, subjects with evening chronotype have previously been reported to be more likely to have obesity and an unhealthy lifestyle [12]. In this regard, in a cross-sectional study including 172 middle-aged adults, the lower the chronotype score, the higher the BMI values in the entire population, suggesting that obesity was a common finding in subjects with evening chronotype. Additionally, in this study, subjects with evening chronotype followed an unhealthier lifestyle (less regular activity and more frequently smokers) than other chronotype categories [12].

In our population, evening chronotype was significantly associated with a higher prevalence of IR subjects (HoMA-IR cut off > 2.5) than the other two chronotype categories. In addition, chronotype score was inversely associated with fasting plasma glucose, insulin levels, and HoMA-IR. Evening chronotype involves a misalignment of physiological circadian rhythms [10] and it has already been reported that humans develop impaired glucose tolerance when subjected to circadian misalignment conditions [10,26,27,28]. As PCOS is already a risk factor for IR, evening chronotype may have an additional deleterious effect on the metabolic profile. In this regard, mutations in multiple clock genes in humans have been shown to contribute to genetic susceptibility to obesity, IR, and type 2 diabetes [10,26,27,28]. Indeed, the risk of developing impaired fasting plasma glucose has been associated with polymorphisms in CRY2 [10,26,28] while the polymorphism in ARNTL has been reported to increase the susceptibility to develop type 2 diabetes [27]. Similarly, having polymorphism in NR1D1 has been associated with the risk of developing obesity [29]. Based on these findings, several studies investigated gene-behavior interactions and showed that interactions between diet and clock gene mutations affect fasting plasma glucose [30], IR [31], body weight [32], and type 2 diabetes [27].

For the first time, we report that women with PCOS with evening chronotype had the lowest adherence to the MD and poorer eating habits. In addition, the lower the chronotype score, the lower the PREDIMED score in these women. Considering the nutritional parameters, women with PCOS with evening chronotype consumed more total energy intake, total and simple carbohydrates, total fat, SFA, PUFA, n-6 PUFAs, and less fiber than women with PCOS with other chronotypes. In addition, regarding the dietary components included in the PREDIMED questionnaire, we found that women with PCOS with evening chronotype consumed less EVOO, red wine, legumes, fish/seafood, and tree nuts and more red/processed meat than women with PCOS with neither and morning chronotypes.

In agreement with our results, it has been reported that subjects with obesity and evening chronotype had the lowest adherence to the MD [12]. In fact, in the study previously mentioned, it has been also reported that evening chronotype had the highest percentage of subjects with low adherence to the MD and the lowest percentages of subjects with average and high adherence to the MD [12].

The association of chronotype categories with the adherence to the MD might be mediated by the food cluster enclosed in the MD. In fact, a high adherence to the MD could also contribute to normal sleeping pattern [33,34] that in turn plays an important role in determining the chronotype [10]. In this regard, in a cross-sectional study of 172 middle-aged adults with obesity, good sleepers (assessed by Pittsburgh Sleep Quality Index < 5) when compared to poor sleepers had significantly higher adherence to the MD [34]. In another cross-sectional study of 1936 individuals, for every point by which the MD score (MEDI-LITE score) increased, the probability of having adequate sleep quality increased by 10% [33].

It appears that eating patterns change in chronotype categories and this has already been reported previously [35,36]. In agreement with this, it has already been reported that individuals with evening chronotype tend to have a less healthy diet, with higher intakes of sugary drinks and chocolate and lower intakes of vegetables, fish, and fruit than individuals with morning chronotype [35,36]. Mota et al., in a cross-sectional study in medical residents (*n* = 72) evaluating chronotype and dietary patterns, found that chronotype score was negatively associated with cholesterol and sweet intake [35]. The same finding was then also confirmed in another cross-sectional study carried out in pregnant women (*n*= 100) [36]. In these women, the morning chronotype was associated with better diet quality [36]. Furthermore, another cross-sectional study reported that a low intake of micronutrients (vitamins and minerals) was also associated with the evening chronotype [37]. In particular, a significant association of the evening chronotype with a lower intake of protein, micronutrients (such as calcium, magnesium, zinc, and vitamins), and vegetables, and a higher intake of noodles has been detected in 112 young women [37]. A deficit of micronutrients such as vitamin D has also been often detected in women with PCOS [6,38] and it has been associated with sleep disturbances [39], a common finding in evening chronotype.

Preference for consumption of specific foods and more generally different dietary patterns may also play a role in worsening normal circadian rhythms [40]. In this regard, it has been reported that the macronutrient composition of the diet can alter the function of central and peripheral circadian clocks in humans [36]. A well-known characteristic of the MD is high fiber content and consequently a low glycemic index (GI) [41]. Our data showed that women with PCOS with evening chronotype consumed more carbohydrates and less fiber than the same women with other two chronotype categories thus suggesting that the diet of these women is characterized by a high GI. Similarly, a recent study by Gangwish et al. carried out in a population of postmenopausal women (*n* = 53,069) found that high GI diets may be a risk factor for insomnia, whereas the intake of unprocessed, fiber-rich, low GI whole-grain carbohydrates such as whole grains reduced this risk [42]. Hyperinsulinemia, resulting from high glycemic load dietary intake, could result in reactive hypoglycemia, thus triggering the secretion of autonomous counter-regulatory hormones such as adrenaline and cortisol, which may cause symptoms such as heart palpitations, tremors, anxiety, and irritability causing waking from sleep [42]. Indeed, St-Onge et al. observed that a low fiber intake and a high intake of SFA and sugars in normal weight adults (*n* = 26) was associated with less restorative sleep resulting in reduced overall sleep quality [43]. In addition, high GI food predisposes to obesity, which is a well-known risk factor for sleep disturbances [44]. Therefore, it is possible that a high-fiber diet such as the MD may be a useful tool for improving sleep in individuals with sleep disturbances [43]. Furthermore, in a cross-sectional study of non-shift workers (*n* = 4435) conducted by Tanaka et al., the association among carbohydrate, lipid, and protein intake and insomnia symptoms, including difficulty initiating sleep, difficulty maintaining sleep, and poor quality of sleep was assessed [45]. This study showed that protein and carbohydrate intake were associated with insomnia; in particular, a low protein intake (19% of energy from protein) and low carbohydrate intake (50% of energy from carbohydrate) were associated with difficulty maintaining sleep. This can probably be explained by the fact that a low protein intake could lead to a low availability of tryptophan, a precursor of serotonin and melatonin, which are well known hormones promoting sleep, while a high protein intake can reduce tryptophan concentrations in the brain due to competition for the blood–brain barrier transporter with other neutral amino acids, causing in both cases symptoms of insomnia [45]. The MD contains various dietary sources of tryptophan such as milk, turkey, chicken, fish, eggs, beans, cheese, and green leafy vegetables. Thus, low adherence to the MD, as in the case of women with PCOS with evening chronotype, results in poor intake of tryptophan-rich foods. The intake of tryptophan rich food has been associated with an improvement of primary insomnia in healthy subjects (*n* = 49) [46]. In this study, administration of food bars containing small dose (250 mg) of tryptophan achieved significant results in reducing waking time during the night, increasing sleep efficiency, and increasing subjective sleep quality [46]. Furthermore, the depletion of tryptophan resulted in sleep fragmentation, rapid eye movement sleep latency and rapid eye movement density compared to baseline and placebo [47,48]. Consumption of tryptophan-rich proteins (e.g., milk protein) may also improve sleep quality by influencing changes in core temperature [49].

The MD also includes a substantial consumption of fruits and clinical studies suggested sleep-promoting effects of some fruits [45,46,47,48]. In humans, consumption of two kiwis one hour before bedtime improved sleep onset, duration, and efficiency in healthy adults (*n* = 24) during a 4-week open clinical trial [50]. In addition, a double-blind pilot study showed that fresh tartar cherry juice reduced insomnia in elderly subjects (*n* = 15) [51]. The effects of cherries have also been reported to increase sleep duration and to reduce the number of awakenings, as measured by actigraphy in Spanish participants (*n* = 12) [52]. These benefits may be facilitated by dietary polyphenols, which have been shown to modulate circadian rhythms [53] and sleep–wake cycles in rodents [54]. According to another hypothesis, these results (on improvement of sleep quality) could probably be due to the high content of antioxidants in fruit. In fact, antioxidants may have beneficial effects in subjects with sleep disorders as they may develop oxidative stress due to the accumulation of free radicals [55]. During sleep, the antioxidant activity increases in the whole body, resulting in the elimination of oxygen free radicals accumulated during wakefulness [55]. In addition to fruit, both polyphenols and antioxidants are also present in other foods that are typical of the MD such as EVOO and red wine that were less consumed in our population of women with PCOS with evening chronotype. In particular, it has been identified that several polyphenols, such as resveratrol, a polyphenol found in red wine, act as dietary activators of Sirtuin 1 (SIRT1) [56]. In turn, SIRT1 modulates transcription factors including period circadian clock 2 (PER2), which are circadian clock genes that in turn regulate the daily rhythms of locomotor activity, metabolism, and behavior [57]. Additionally, SIRT1 modulates the ventromedial hypothalamic clock, a brain region that contains neuronal food-synchronized clocks contributing to regulation of the circadian rhythm in feeding behavior [57]. Thus, it is possible to speculate that a high adherence to the MD and more specifically the consumption of certain foods with well-known positive effects on sleep may contribute to the maintenance of normal circadian rhythms and therefore have an influence on chronotype [10].

The main strength of our study was the novelty of highlighting the role of chronotype assessment in women with PCOS. Identifying women with PCOS and evening chronotype becomes of paramount importance because they might be at risk of following unhealthy diet thus being at high risk of developing metabolic diseases. In this context, using a tool as easy as the MEQ questionnaire, women with PCOS with a worse clinical and metabolic picture could be identified early and thus could be tackled with more tailored diet plans. Indeed, an intervention that seeks to align the attitude of the evening chronotype with the circadian time could be an early attempt to improve the metabolic status of women with PCOS. The limitation of the study is represented above all by the cross-sectional experimental design, which, although showing the association of the evening chronotype with a worsening of IR in women with PCOS, fails to provide any explanation of the causality of this association. It is recommended that future longitudinal studies investigate the causality among these variables with particular emphasis on the implementation of lifestyle interventions. Although a CLOCK-gene assessment of chronotype should be more accurate but more difficult to carry out, there are several studies that validated the association of chronotype, and thus the assessment of chronotype categories through a questionnaire, with CLOCK-gene based assessed of chronotype [58]. This allows us to use this tool for the assessment of chronotype in our study. Finally, both genetic and environmental factors, including nutrition and gut microbiota, influence the distribution of chronotypes. However, we did not include the gut microbiota or gut-derived metabolites, including trimethylamine N-oxide, in this study [17].

## 5. Conclusions

In summary, the current study presented the first evidence that women with PCOS with evening chronotype have worse anthropometric measurements, more severe IR, and in general follow an unhealthier lifestyle than women with PCOS with other chronotype categories. These same women have also the lowest adherence to the MD and poorest eating habits. Our results support the evidence that chronotype assessment could be a new and effective tool to screen the eating habits, and more generally the lifestyle, of women with PCOS that could be used by multidisciplinary team that usually take care of women with PCOS such as gynecologists, endocrinologists, and dietitians. Thus, chronotype assessment could be a preliminary examination to be performed in women with PCOS in order to drive the most appropriate nutritional approach.

## Figures and Tables

**Table 1:** BMI categories, lifestyle habits, and HoMA-IR of women with PCOS according to chronotype categories.

Parameters	Morning Chronotype *n* = 31, 27.7%		Neither Chronotype *n* = 33, 29.5%		Evening Chronotype *n* = 48, 42.9%		χ^2^	*p*-Value
BMI categories								
Normal weight (*n*, %)	22	71.0	2	6.1	0	0	62.91	**<0.001**
Overweight (*n*, %)	4	12.9	18	54.5	7	14.6	20.04	**<0.001**
Grade I obesity (*n*, %)	1	3.2	6	18.2	17	35.4	11.89	**0.003**
Grade II obesity (*n*, %)	4	12.9	7	21.2	24	50.0	19.16	**0.001**
Physical activity								
YES (*n*, %)	20	64.5	13	39.4	3	6.3	30.45	**<0.001**
NO (*n*, %)	11	35.5	20	60.6	45	93.8		
Smoking								
YES (*n*, %)	2	6.5	2	6.1	25	52.1	30.03	**<0.001**
NO (*n*, %)	29	93.5	31	93.9	23	47.9		
HoMA-IR								
<2.5 (*n*, %)	30	96.8	26	78.8	19	39.6	30.81	**<0.001**
≥2.5 (*n*, %)	1	3.2	7	21.2	29	60.4		

A *p* value in bold type denotes a significant difference (*p* < 0.05). BMI, Body Mass Index; HoMA-IR, homeostasis model assessment-insulin resistance.

**Table 2:** Age, BMI, WC. HoMA-IR, and adherence to the MD in chronotype categories in comparison.

Parameters	Mean ± SD	*p*-Value
Age (years)		0.763 *
Evening vs. morning chronotype	24.58 ± 5.16 vs. 24.23 ± 6.49	1.000
Evening vs. neither chronotype	24.58 ± 5.16 vs. 23.67 ± 4.95	1.000
Neither vs. morning chronotype	23.67 ± 4.95 vs. 24.23 ± 6.49	1.000
BMI (kg/m^2^)		<0.001 *
Evening vs. morning chronotype	34.92 ± 3.87 vs. 25.95 ± 4.66	**<0.001**
Evening vs. neither chronotype	34.92 ± 3.87 vs. 29.91 ± 4.44	**<0.001**
Neither vs. morning chronotype	29.91 ± 4.44 vs. 25.95 ± 4.66	**<0.001**
WC (cm)		<0.001 *
Evening vs. morning chronotype	110.71 ± 15.95 vs. 89.33 ± 12.32	**<0.001**
Evening vs. neither chronotype	110.71 ± 15.95 vs. 98.15 ± 11.11	**<0.001**
Neither vs. morning chronotype	98.15 ± 11.11 vs. 89.33 ± 12.32	**0.034**
HoMA-IR		<0.001 *
Evening vs. morning chronotype	4.85 ± 4.26 vs. 0.96 ± 1.69	**<0.001**
Evening vs. neither chronotype	4.85 ± 4.26 vs. 1.9 ± 2.29	**<0.001**
Neither vs. morning chronotype	1.9 ± 2.29 vs. 0.96 ± 1.69	0.709
PREDIMED score		<0.001 *
Evening vs. morning chronotype	5.06 ± 1.93 vs. 9.67 ± 1.98	**<0.001**
Evening vs. neither chronotype	5.06 ± 1.93 vs. 7.21 ± 1.95	**<0.001**
Neither vs. morning chronotype	7.21 ± 1.95 vs. 9.67 ± 1.98	**<0.001**

A *p* value in bold type denotes a significant difference (*p* < 0.05). Results are expressed as the mean ± SD. BMI, Body Mass Index; HoMA-IR, homeostasis model assessment-insulin resistance; PREDIMED, Prevención con Dieta Mediterránea; and SD, standard deviation. * Differences among three groups.

**Table 3:** Differences in nutritional parameters of women with PCOS, according to chronotype categories.

Parameters	Mean ± SD	*p*-Value
Total energy intake (kcal)		<0.001 *
Evening vs. morning chronotype	2372.77 ± 301.32 vs. 2053.19 ± 198.04	**<0.001**
Evening vs. neither chronotype	2372.77 ± 301.32 vs. 2240.39 ± 250.94	0.082
Neither vs. morning chronotype	2240.39 ± 250.94 vs. 2053.19 ± 198.04	**0.015**
Carbohydrate (g of total kcal)		<0.001 *
Evening vs. morning chronotype	326.40 ± 45.05 vs. 280.71 ± 28.54	**<0.001**
Evening vs. neither chronotype	326.40 ± 45.05 vs. 306.79 ± 33.94	0.072
Neither vs. morning chronotype	306.79 ± 33.94 vs. 280.71 ± 28.54	**0.021**
Complex (g of total kcal)		0.502 *
Evening vs. morning chronotype	174.37 ± 30.59 vs. 170.23 ± 19.99	1.000
Evening vs. neither chronotype	174.37 ± 30.59 vs. 177.73 ± 21.61	1.000
Neither vs. morning chronotype	177.73 ± 21.61 vs. 170.23 ± 19.99	0.726
Simplex (g of total kcal)		<0.001 *
Evening vs. morning chronotype	152.03 ± 34.06 vs. 110.48 ± 20.37	**<0.001**
Evening vs. neither chronotype	152.03 ± 34.06 vs. 129.07 ± 29.53	**0.002**
Neither vs. morning chronotype	129.07 ± 29.53 vs. 110.48 ± 20.37	**0.040**
Fiber (g/day)		<0.001 *
Evening vs. morning chronotype	13.55 ± 2.99 vs. 17.91 ± 3.61	**<0.001**
Evening vs. neither chronotype	13.55 ± 2.99 vs. 15.88 ± 3.06	**0.004**
Neither vs. morning chronotype	15.88 ± 3.06 vs. 17.91 ± 3.61	**0.037**
Protein (g of total kcal)		0.403 *
Evening vs. morning chronotype	85.86 ± 8.89 vs. 86.65 ± 9.27	1.000
Evening vs. neither chronotype	85.86 ± 8.89 vs. 88.93 ± 12.43	0.552
Neither vs. morning chronotype	88.93 ± 12.43 vs. 86.65 ± 9.27	1.000
Fat (g of total kcal)		<0.001 *
Evening vs. morning chronotype	80.41 ± 14.01 vs. 64.86 ± 10.01	**<0.001**
Evening vs. neither chronotype	80.41 ± 14.01 vs. 73.05 ± 10.74	**0.025**
Neither vs. morning chronotype	73.05 ± 10.74 vs. 64.86 ± 10.01	**0.024**
SFA (g of total kcal)		<0.001 *
Evening vs. morning chronotype	29.27 ± 6.61 vs. 18.40 ± 5.25	**<0.001**
Evening vs. neither chronotype	29.27 ± 6.61 vs. 23.48 ± 5.88	**<0.001**
Neither vs. morning chronotype	23.48 ± 5.88 vs. 18.40 ± 5.25	**0.003**
Unsaturated fat (g of total kcal)		0.060 *
Evening vs. morning chronotype	51.15 ± 9.81 vs. 46.46 ± 6.96	0.055
Evening vs. neither chronotype	51.15 ± 9.81 vs. 49.57 ± 7.65	1.000
Neither vs. morning chronotype	49.57 ± 7.65 vs. 46.46 ± 6.96	0.438
MUFA (g of total kcal)		0.007 *
Evening vs. morning chronotype	36.95 ± 5.09 vs. 38.14 ± 3.43	0.728
Evening vs. neither chronotype	36.95 ± 5.09 vs. 40.14 ± 4.12	**0.005**
Neither vs. morning chronotype	40.14 ± 4.12 vs. 38.14 ± 3.43	0.214
PUFA (g of total kcal)		<0.001 *
Evening vs. morning chronotype	14.20 ± 7.53 vs. 8.32 ± 5.63	**<0.001**
Evening vs. neither chronotype	14.20 ± 7.53 vs. 9.42 ± 5.12	**0.004**
Neither vs. morning chronotype	9.42 ± 5.12 vs. 8.32 ± 5.63	1.000
n-3 PUFA (g/day)		0.483 *
Evening vs. morning chronotype	3.29 ± 2.39 vs. 3.03 ± 2.20	1.000
Evening vs. neither chronotype	3.29 ± 2.39 vs. 3.70 ± 2.06	1.000
Neither vs. morning chronotype	3.70 ± 2.06 vs. 3.03 ± 2.20	0.705
n-6 PUFA (g/day)		<0.001 *
Evening vs. morning chronotype	10.91 ± 7.65 vs. 5.29 ± 5.53	**0.001**
Evening vs. neither chronotype	10.91 ± 7.65 vs. 5.72 ± 4.72	**0.001**
Neither vs. morning chronotype	5.72 ± 4.72 vs. 5.29 ± 5.53	1.000

A *p* value in bold type denotes a significant difference (*p* < 0.05). Results are expressed as the mean ± SD. SFA, Saturated Fatty Acids; MUFA, MonoUnsaturated Fatty Acids; PUFA, PolyUnsaturated Fatty Acids; and SD, standard deviation. * Differences among three groups.

**Table 4:** Response frequency of dietary components included in the PREDIMED questionnaire of women with PCOS, according to chronotype categories.

Questions PREDIMED Questionnaire	Morning Chronotype *n* = 31, 27.7%		Neither Chronotype *n* = 33, 29.5%		Evening Chronotype *n* = 48, 42.9%			
	*n*	%	*n*	%	*n*	%	χ^2^	*p*-Value
Use of EVOO as main culinary lipid	29	93.5	29	87.9	30	62.5	17.20	**0.001**
EVOO > 4 tablespoons	24	77.4	18	54.5	22	45.8	3.51	0.173
Vegetables ≥ 2 servings/day	25	80.6	17	51.5	12	25.0	1.55	0.460
Fruits ≥ 3 servings/day	26	83.9	21	63.6	20	41.7	1.12	0.099
Red/processed meats < 1/day	13	41.9	22	66.7	27	56.3	29.87	**<0.001**
Butter, cream, margarine < 1/day	15	48.4	16	48.5	16	33.3	4.02	0.134
Soda drinks < 1/day	15	48.4	22	66.7	16	33.3	10.16	**0.006**
Wine glasses ≥ 7/week	19	61.3	4	12.1	7	14.6	7.91	**0.019**
Legumes ≥ 3/week	20	64.5	18	54.5	22	45.8	6.52	**0.038**
Fish/seafood ≥ 3/week	28	90.3	7	21.2	0	0	32.03	**<0.001**
Commercial sweets and confectionery ≤ 2/week	23	74.2	20	60.6	16	33.3	1.28	0.527
Tree nuts ≥ 3/week	19	61.3	5	15.2	7	14.6	6.39	**0.041**
Poultry more than red meats	26	83.9	19	57.6	18	37.5	0.15	0.928
Use of sofrito sauce ≥ 2/week	18	58.1	20	60.6	30	62.5	1.58	0.466

A *p* value in bold type denotes a significant difference (*p* < 0.05). PREDIMED, Prevención con Dieta Mediterránea; EVOO, extra virgin olive oil.

**Table 5:** Correlations of chronotype score with studied parameters in women with PCOS.

Parameters	Chronotype Score *n* = 112	
	r	*p*-Value
Age (years)	0.006	0.951
Anthropometric measurement		
BMI (kg/m^2^)	−0.775	**<0.001**
WC (cm)	−0.673	**<0.001**
Metabolic, hormonal, and inflammatory profile		
Fasting plasma glucose (mg/dL)	−0.486	**<0.001**
Insulin levels (µU/mL)	−0.568	**<0.001**
HoMA-IR	−0.556	**<0.001**
Nutritional parameters		
PREDIMED score	0.803	**<0.001**
Total energy intake (kcal)	−0.507	**<0.001**
Carbohydrate (g of total kcal)	−0.563	**<0.001**
Complex (g of total kcal)	−0.116	0.221
Simple (g of total kcal)	−0.609	**<0.001**
Fiber (g/day)	0.531	**<0.001**
Protein (g of total kcal)	0.053	0.576
Fat (g of total kcal)	−0.598	**<0.001**
SFA (g of total kcal)	−0.711	**<0.001**
Unsaturated fat (g of total kcal)	−0.324	**<0.001**
MUFA (g of total kcal)	0.088	0.356
PUFA (g of total kcal)	−0.465	**<0.001**
n-3 PUFA (g/day)	−0.035	0.712
n-6 PUFA (g/day)	−0.456	**<0.001**

A *p* value in bold type denotes a significant difference (*p* < 0.05). BMI, Body Mass Index; WC, waist circumference; HoMA-IR, homeostasis model assessment-insulin resistance; PREDIMED, Prevención con Dieta Mediterránea; SFA, Saturated Fatty Acids; MUFA, MonoUnsaturated Fatty Acids; and PUFA, PolyUnsaturated Fatty Acids.

**Table 6:** Bivariate proportional OR models performed to assess the association of chronotype score with the dietary components included in the PREDIMED questionnaire and with PREDIMED categories.

Parameters	Chronotype Score			
	OR	*p*-Value	95% IC	R^2^
Use of EVOO as main culinary lipid	1.08	**<0.001**	1.04–1.12	0.19
EVOO > 4 tablespoons	1.04	**0.001**	1.02–1.07	0.10
Vegetables ≥ 2 servings/day	1.06	**<0.001**	1.04–1.09	0.21
Fruits ≥ 3 servings/day	1.05	**<0.001**	1.03–1.08	0.15
Red/processed meats < 1/day	0.99	0.672	0.98–1.02	0.01
Butter, cream, margarine < 1/day	1.02	0.076	0.99–1.04	0.03
Soda drinks < 1/day	1.03	**0.010**	1.01–1.05	0.06
Wine glasses ≥ 7/week	1.05	**<0.001**	1.03–1.08	0.14
Legumes ≥ 3/week	1.03	**0.019**	1.00–1.05	0.05
Fish/seafood ≥ 3/week	1.29	**<0.001**	1.15–1.14	0.57
Commercial sweets and confectionery ≤ 2/week	1.05	**<0.001**	1.03–1.08	0.17
Tree nuts ≥ 3/week	1.06	**<0.001**	1.03–1.09	0.18
Poultry more than red meats	1.04	**<0.001**	1.02–1.08	0.16
Use of sofrito sauce ≥ 2/week	0.99	0.769	0.98–1.02	0.01

A *p* value in bold type denotes a significant difference (*p* < 0.05). EVOO, extra virgin olive oil.

## Data Availability

Results attained in this study are included in the manuscript. Individual data are not publicly available due to ethical restrictions.
